# Water-separated part of *Chloranthus serratus* alleviates lipopolysaccharide- induced RAW264.7 cell injury mainly by regulating the MAPK and Nrf2/HO-1 inflammatory pathways

**DOI:** 10.1186/s12906-019-2755-6

**Published:** 2019-12-02

**Authors:** Shuping Sun, Yunyan Du, Chuanliu Yin, Xiaoguo Suo, Rui Wang, Rongping Xia, Xiaoping Zhang

**Affiliations:** 1 College of Life Science, Anhui Normal University, Wuhu, 241000 Anhui China; 2 College of Pharmacy, Wannan Medical College, Wuhu, 241002 Anhui China; 3 Institute of Natural Daily Chemistry, Wannan Medical College, Wuhu, 241002 Anhui China

**Keywords:** *Chloranthus serratus*, LPS, RAW264.7 cells, Anti-inflammatory, MAPK pathway, NF-κB pathway

## Abstract

**Background:**

*Chloranthus serratus* (Chloranthaceae) has been used to treat bruises, rheumatoid and bone pain. However, the anti-inflammatory mechanisms of *C. serratus* in vitro have not been fully elucidated. The present study aimed to explore the anti-inflammatory activity and potential mechanisms of *C. serratus*’s separated part of water (CSSPW) in lipopolysaccharide (LPS)-induced RAW264.7 cells.

**Methods:**

The concentrations of CSSPW were optimized by CCK-8 method. Nitric oxide (NO) content was detected by one-step method. The levels of inflammatory cytokines were determined by enzyme-linked immunosorbent assay (ELISA). Gene expression of inducible nitric oxide synthase (iNOS) and cyclooxygenase-2 (COX-2) was detected by real-time quantitative PCR (qPCR). Immunofluorescence and DCFH-DA fluorescent probes were used to detect p65 nuclear translocation and reactive oxygen species (ROS) content, respectively. Western blotting was used to assay the protein expression of mitogen-activated protein kinases (MAPK), nuclear factor-kappa B (NF-κB) and nuclear transcription factor E2 related factor 2/haem oxygenase-1 (Nrf2/HO-1) pathways.

**Results:**

The final concentrations of 15 ng/mL, 1.5 μg/mL and 150 μg/mL were selected as low, medium and high doses of CSSPW, respectively. CSSPW treatment significantly reduced the generation of NO, tumour necrosis factor-α (TNF-α), interleukin-6 (IL-6), prostaglandinE_2_ (PGE_2_), iNOS mRNA and COX-2 mRNA in response to LPS stimulation. Furthermore, the protein expression of the MAPK and NF-κB pathways was suppressed by CSSPW treatment, as well as p65 nuclear translocation and ROS production. In contrast, the protein expression of the Nrf2/HO-1 pathway was markedly upregulated.

**Conclusions:**

CSSPW exerts its anti-inflammatory effect via downregulating the production of pro-inflammatory mediators, inhibiting the activation of NF-κB and MAPK pathways, as well as activating Nrf2/HO-1 pathway in LPS-induced RAW264.7 cells.

## Background

Inflammation is a defence response against a variety of harmful stimuli (e.g., tissue injury, infection and stimulants), which is also the most common pathological process in human diseases [1, 2]. Inflammation is generally divided into acute and chronic inflammation, which can lead to a series of inflammatory diseases such as rheumatoid arthritis and gastritis. If not treated in time, inflammation may further develop into various cancers [3, 4].

The occurrence of inflammation is accompanied by the production of inflammatory mediators such as prostaglandin E_2_ (PGE_2_), interleukin-6 (IL-6), tumour necrosis factor-α (TNF-α) and nitric oxide (NO). The over-expression of these inflammatory mediators may cause serious damage to cells and tissues, even resulting in organ failure [3, 5]. Moreover, two pro-inflammatory enzymes, inducible nitric oxide synthase (iNOS) and cyclooxygenase-2 (COX-2), play synergistic roles in inflammatory diseases, and drugs with dual inhibitory effects on iNOS and COX-2 mRNA have great anti-inflammatory potential [6]. Hence, inhibiting the over-expression of related inflammatory mediators is an effective method to control inflammation.

At present, there are many anti-inflammatory chemical-based drugs, such as indomethacin, prednisone, ibuprofen and aspirin. However, these drugs can damage the kidneys [7]. Fortunately, traditional Chinese medicine (TCM) provides an alternative or better options for patients with inflammation. TCM has good anti-inflammatory and immunological activity, which is used to treat inflammation with multi-component, multi-link and multi-target characteristics [8]. Therefore, it is of great significance to develop natural anti-inflammatory drugs.

*Chloranthus serratus* (Thunb.) Roem. et Schalt (Chloranthaceae family), mainly produced in Anhui, Zhejiang, Guangxi, Yunnan and other provinces of China, can promote blood circulation, prevent inflammation and other diseases [9]. According to the records in the *Modern Chinese Pharmacy Dictionary*, *C. serratus* can relax muscles and joints, reduce swelling, relieve pain, treat bruises, fractures, rheumatism, etc. [10]. Its root is often used as medicine and has obvious anti-inflammatory activity in vivo [11]. Our previous study has shown that *C. serratus*’s separated part of water (CSSPW) has anti-inflammatory activity in rats. However, the anti-inflammatory mechanisms of CSSPW in lipopolysaccharide (LPS)-induced RAW264.7 cells remain unclear.

This study aimed to investigate the anti-inflammatory effects and potential mechanisms of CSSPW on LPS-stimulated RAW264.7 cells. We determined the production of corresponding inflammatory factors and mRNA levels of pro-inflammatory mediators. Finally, the levels of related signal pathways were detected to clarify the anti-inflammatory mechanisms.

## Methods

### Reagents

The RAW264.7 cells (TCM13) were purchased from the American Type Culture Collection (Rockville, MD, USA). LPS was obtained from Beijing Solarbio Co., Ltd. (Beijing, China). CCK-8 kit was purchased from Jiangsu Kaiji Biotechnology Co., Ltd. (Jiangsu, China). NO was obtained from Jiancheng Biotechnology Co., Ltd. (Nanjing, China). TNF-α, IL-6 and PGE_2_ enzyme linked immunosorbent assay (ELISA) kits were provided by Shanghai Youchu Trading Co., Ltd. (Shanghai, China). Nuclear transcription factor E2 related factor 2 (Nrf2), p65 and β-tubulin antibodies were obtained from Proteintech Group, Inc. (USA). P-p65 and p-p38antibodies were obtained from Affinity Biosciences (USA). ERK1/2, p-ERK1/2, p38, p-JNK, JNK antibodies and DCFH-DA kit were purchased from Beyotime Biotechnology Co., Ltd. (Shanghai, China). β-actin, heme oxygenase-1 (HO-1) and IgG antibodies were purchased from Boster Biological Engineering Co., Ltd. (Wuhan, China). GAPDH, iNOS and COX-2 primers were obtained from Servicebio Biotechnology Co., Ltd. (Wuhan, China), etc.

### Sample preparation

*C. serratus* was harvested in Yulin (Guangxi, China). Referring to *the Modern Chinese Pharmacy Dictionary* [10], it was determined to be genuine by Professor Zhu JH of Wannan Medical College, and a voucher specimen of the plant (ANUB14096) was deposited in the Herbarium Centre, Anhui Normal University, China. After washing and drying naturally, the root was separated and crushed to coarse powder, which was stored at room temperature.

The coarse powder of the *C. serratus* root was immersed into 12 times 75% ethanol for 0.5 h and refluxed for 1.5 h, then extracted with 10 times and 8 times 75% ethanol for 1 h, respectively. The tertiary filtrate was combined and the solvent was recovered at low pressure until alcohol free, then extracted successively with an equal amount of chloroform, ethyl acetate and n-butanol for three times. The residual phase after extraction by n-butanol was the aqueous phase. Subsequently, the aqueous phase was concentrated, dried and crushed to obtain the water-separated part, which was stored at room temperature in a desiccator. Extraction rate (%) = mass of the water-separated part (g) / mass of coarse powder from the root (g) × 100%, which was 1.94%.

### Cell culture and viability assay

RAW264.7 cells were cultured in high glucose Dulbecco’s modified Eagle’s medium (DMEM) containing 10% (v/v) foetal bovine serum (FBS, HyClone, USA) and 1% penicillin-streptomycin at 37 °C in a humidified atmosphere with 5% CO_2_. The medium was replaced every 2 days. Cells in their logarithmic growth stage were chosen for follow-up experiments.

Cell viability was detected by CCK-8 method. The cells were seeded in 96-well culture plates (2 × 10^4^ cells/well) and treated with 100 μL of different concentrations of CSSPW (0, 11.5, 23, 46, 92, 184, 368, 736 and 1472 μg/mL) and LPS (0, 0.5, 1.0, 1.5 and 2.0 μg/mL) for 24 h. Then 10 μL of CCK-8 solution was added to each well except the blank group. Next, the 6-well plates were placed in the incubator and cultured for 2 h. OD values were measured at 450 nm wavelength and 600 nm reference wavelength with a microplate reader (Shenzhen Sante Electronics Co., Ltd., Shenzhen, China). Cell viability (%) = (OD_drug group_ - OD_blank group_)/(OD _control group_ - OD _blank group_) × 100%.

### Experimental design

The cells were inoculated into a 6-well plate and divided into control (Con) group, model (LPS) group, dexamethasone-positive (Dex) group, and low-dose, middle-dose and high-dose CSSPW groups. After 1 h of adherent growth, the drug solution was individually added to the corresponding wells and cultured for 0.5 h. Then, 1 mL of LPS solution was added to the wells, except those of the Con group. After inoculation for 24 h, the cell morphology was observed and photographed with an OLYMPUS inverted microscope (Kunshan Nuopusen Laboratory Products Technology Co., Ltd., Kunshan, China).

### Determination of the inflammatory factors NO, TNF-α, IL-6 and PGE_2_

The cells were co-treated with CSSPW (15 ng/mL, 1.5 μg/mL and 150 μg/mL) and LPS (1 μg/mL) for 24 h. After centrifugation, cell-free supernatants were collected for assaying NO, TNF-α, IL-6 and PGE_2_ production. The NO content was measured by one-step method in accordance with the manufacturer’s instructions. The levels of TNF-α, IL-6 and PGE_2_ were determined by ELISA using the automatic biochemical analyser (Hitachi, Japan) according to the protocols of commercial assay kits.

### Real-time quantitative PCR analysis

The expression of COX-2 and iNOS mRNA was detected by real-time fluorescence quantitative PCR. The medium was removed, and the cells were reserved for RNA extraction. The purity of the RNA was determined with a NanoDrop 2000 Ultramicrospectrophotometer (Thermo Fisher Scientific, USA).

The reverse transcription was as follows: RNA solution (2 μL), oligo (dT) 18 (1 μL), and deionized water (9 μL) were added and incubated at 65 °C for 5 min. Then, 5 × reaction buffer (4 μL), 10 mM dNTP mix (2 μL), Ribo Lock RNase inhibitor (1 μL) and RevertAi M-MuLVreverse transcriptase (1 μL) were added. The final solution was incubated at 42 °C for 60 min and then at 70 °C for 5 min to reverse transcribe RNA into cDNA.

The iNOS primers used were 5′-CACCTTGGAAGAGGAGCAACTAC-3′ (Forward) and 5′-GAGCAAAGGCGCAGAACTGA-3′(Reverse). The COX-2 primers used were 5′-ATAGACGAAATCAACAACCCCG-3′ (Forward) and 5′-GGATTGGAAGTTCTATTGGCAG-3′ (Reverse). GAPDH was used as a positive control and the primers used were 5′-CCTCGTCCCGTAGACAAAATG-3′ (Forward) and 5′-TGAGGTCAATGAAGGGGTCGT-3′ (Reverse).

PCR was performed as follows: 95 °C for 10 min; 95 °C for 15 s and 60 °C for 60 s for 40 cycles and 72 °C for 1 min. Finally, the melting curve was obtained. Ct values of the output were quantitatively analysed by the 2^-ΔΔCt^ method.

### Western blotting analysis

The RAW264.7 cells were inoculated in 6-well plates, and the culture medium was discarded after treatment with CSSPW (15 ng/mL, 1.5 μg/mL and 150 μg/mL) and LPS (1 μg/mL) for 24 h. The cells were lysed with lysate (RIPA:PMSF:phosphatase inhibitor = 100:1:1) for 10 min on ice, followed by centrifugation at 12,000 r/min and 4 °C for 20 min. The target proteins were collected and isolated by electrophoresis (Bio-Rad, USA) with 8 and 10% SDS-PAGE, then transferred to nitrocellulose (NC) membranes. After blocking with 5% skim milk at room temperature for 2 h, the NC membranes were incubated with the primary antibodies specific to Nrf2, HO-1, p65, p-p65, ERK, p-ERK, p38, p-p38, JNK, p-JNK, β-actin and β-tubulin at 4 °C overnight, subsequently incubated with the corresponding HRP-conjugated secondary antibodies at room temperature for 1 h. Then the ECL developer was added to expose the bands by Amersham Imager600 Ultra-sensitive Multi-function Imager (General Electric Co., Ltd., USA). Grey values were obtained by ImageJ software to quantitatively analyse protein expression.

### Detection of p65 nuclear transcription

Cover slips were placed in the wells to allow the cells to adhere to them. RAW264.7 cells were inoculated in a 6-well plate, then the medium was discarded after treatment with CSSPW (15 ng/mL, 1.5 μg/mL and 150 μg/mL) and LPS (1 μg/mL) for 24 h. The stimulated RAW264.7 cells were fixed with 4% paraformaldehyde for 30 min, and then permeabilized with 0.2% Triton X-100 for 10 min. After incubation with the blocking solution for 2 h, the cells were incubated with the p65 antibody at 4 °C overnight and then incubated with FITC-labelled goat anti-rabbit IgG at 37 °C for 1 h. Nuclei were stained with DAPI staining agent for 5 min in the dark. Then, the cells were sealed, observed and photographed with a TCSSP8 Laser Confocal Microscope (Leica, Germany).

### Determination of ROS levels

ROS levels were detected by fluorescent probes. DCFH-DA was diluted (1:200) in serum-free medium to a final concentration of 50 μmol/L. After the cells were co-cultured for 24 h with CSSPW (15 ng/mL, 1.5 μg/mL and 150 μg/mL) and LPS (1 μg/mL) in a six-well plate, the cell culture medium was removed, and 1 mL/well of the diluted DCFH-DA was added and incubated at 37 °C for 1 h in the incubator. The cells were washed with serum-free cell culture medium for 3 times to sufficiently remove the DCFH-DA that did not enter the cells. Then, 1 mL PBS was added, and using an excitation wavelength of 488 nm and an emission wavelength of 525 nm, the fluorescence intensity was observed and photographed under an inverted fluorescence microscope. Then the generation rate of ROS was obtained by ImageJ software and analysed quantitatively.

### UV fingerprint analysis

The solution of the sample was obtained by accurately weighing 0.05 g of CSSPW and dissolving it to 1 mg/mL with distilled water. Using Hitachi U-5100 spectrophotometer (Hitachi High-Tech Science Corporation, Japan), blank correction with distilled water, scanning under the following spectral conditions: data mode: Abs; scanning range: 190–500 nm; scanning speed: 400 nm/min; delaying: 0 s; response: fast; sampling interval: 1.0 nm; cycle time: 1.0 min; slit width: 5.00 nm.

### Statistical analysis

For statistical analysis, SPSS17.0 software was employed. All data were expressed as mean ± SD, and one-way analysis of variance was used to compare the means of multiple samples, then, LSD was performed for the post hoc test. *p* < 0.05 indicated a significant difference.

## Results

### Cell viability

**Fig. 1:**
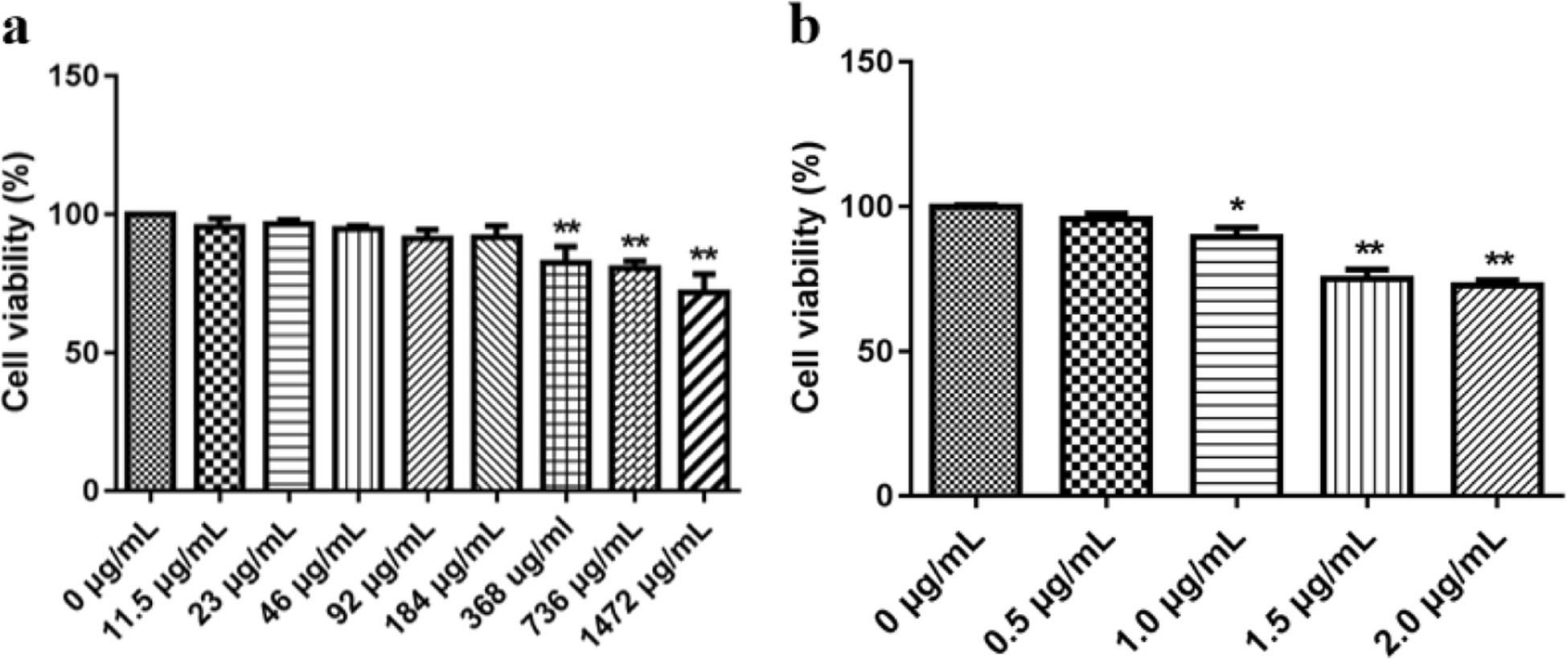
Effects of different concentrations of CSSPW (**a**) and LPS (**b**) on cell viability. All data were represented as mean ± SD (*n* = 6); ^*^p < 0.05, ^**^p < 0.01 vs. the 0 μg/mL

As shown in Fig. 1, the cell viability was almost 100% at CSSPW concentrations of 0–184 μg/mL and LPS concentrations of 0–0.5 μg/mL. The CSSPW concentration in the range of 368–1472 μg/mL and LPS concentrations in the range of 1.5–2.0 μg/mL both had obvious effects on cell viability (*p* < 0.01). CSSPW did not obviously affect cell viability up to 184 μg/mL in the medium. Based on the preliminary results, the experimental concentrations of CSSPW were finally determined to be 15 ng/mL, 1.5 μg/mL and 150 μg/mL, and the experimental concentration of LPS was 1 μg/mL [12].

### Effects on cell morphology

**Fig. 2:**
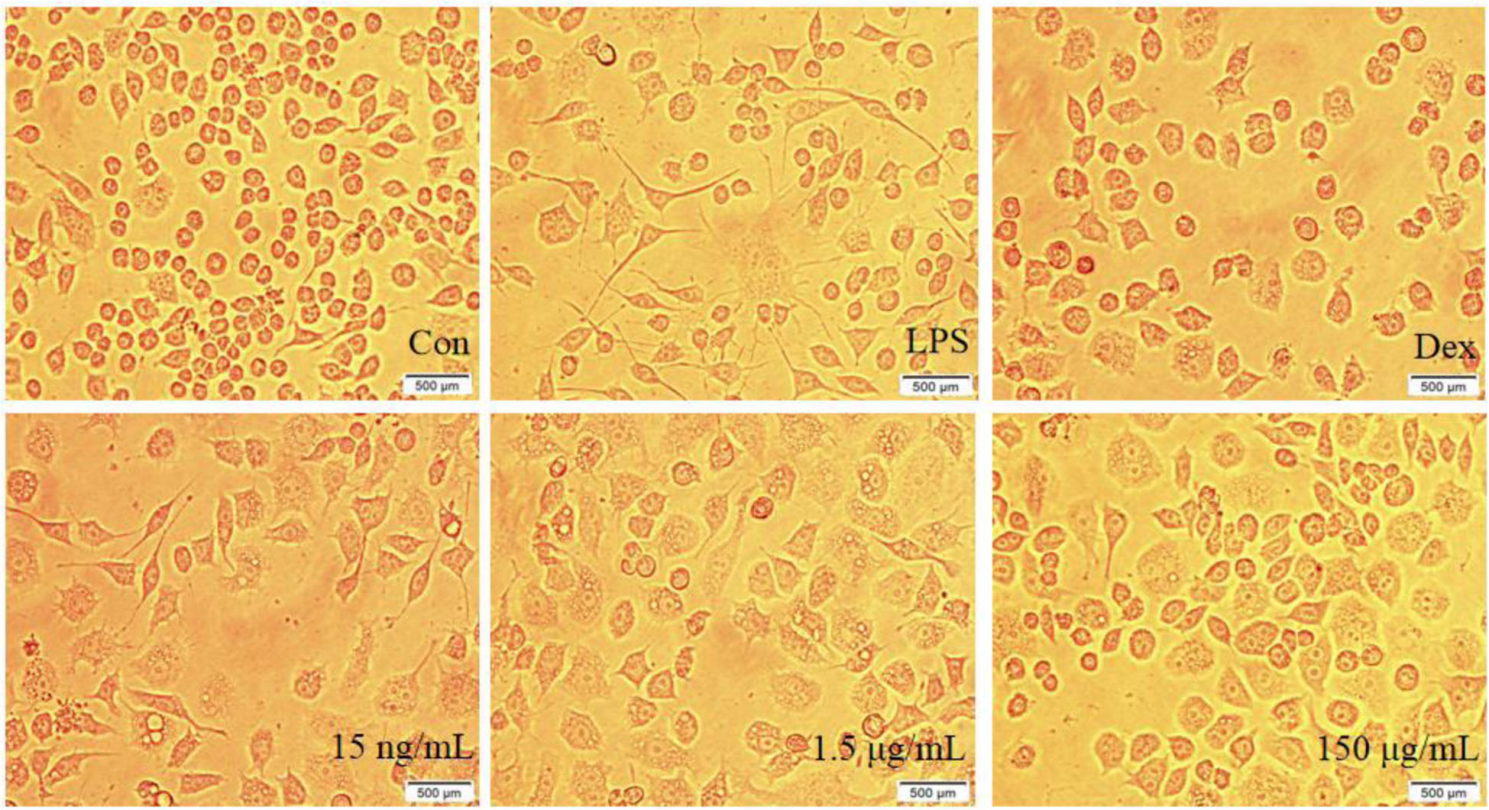
Cell morphology in each group (400 ×). Cells were incubated with CSSPW (15 ng/mL, 1.5 μg/mL and 150 μg/mL), Dex (0.13 mg/mL) and LPS (1 μg/mL) for 24 h. The morphology of cells in the 1.5 μg/mL and 150 μg/mL groups was more regular than that in the LPS and 15 ng/mL groups. These results indicated that CSSPW could alleviate LPS-induced formation of pseudopods, irregular shape and cell vacuoles in a dose-dependent manner

Cell morphology can reflect the health of cells, which is considered one of the noteworthy characteristics of the anti-inflammatory effect. Figure 2 shows that LPS-challenged cells formed more pseudopods of irregular shapes and more cellular vacuoles than those of the Con group, which indicated that LPS stimulation was successful. However, after treatment with CSSPW (15 ng/mL, 1.5 μg/mL and 150 μg/mL), the number of pseudopods, irregular shapes and vacuoles was significantly reduced. The effect was more pronounced at higher concentrations.

### Effects on the production of inflammatory factors

**Fig. 3:**
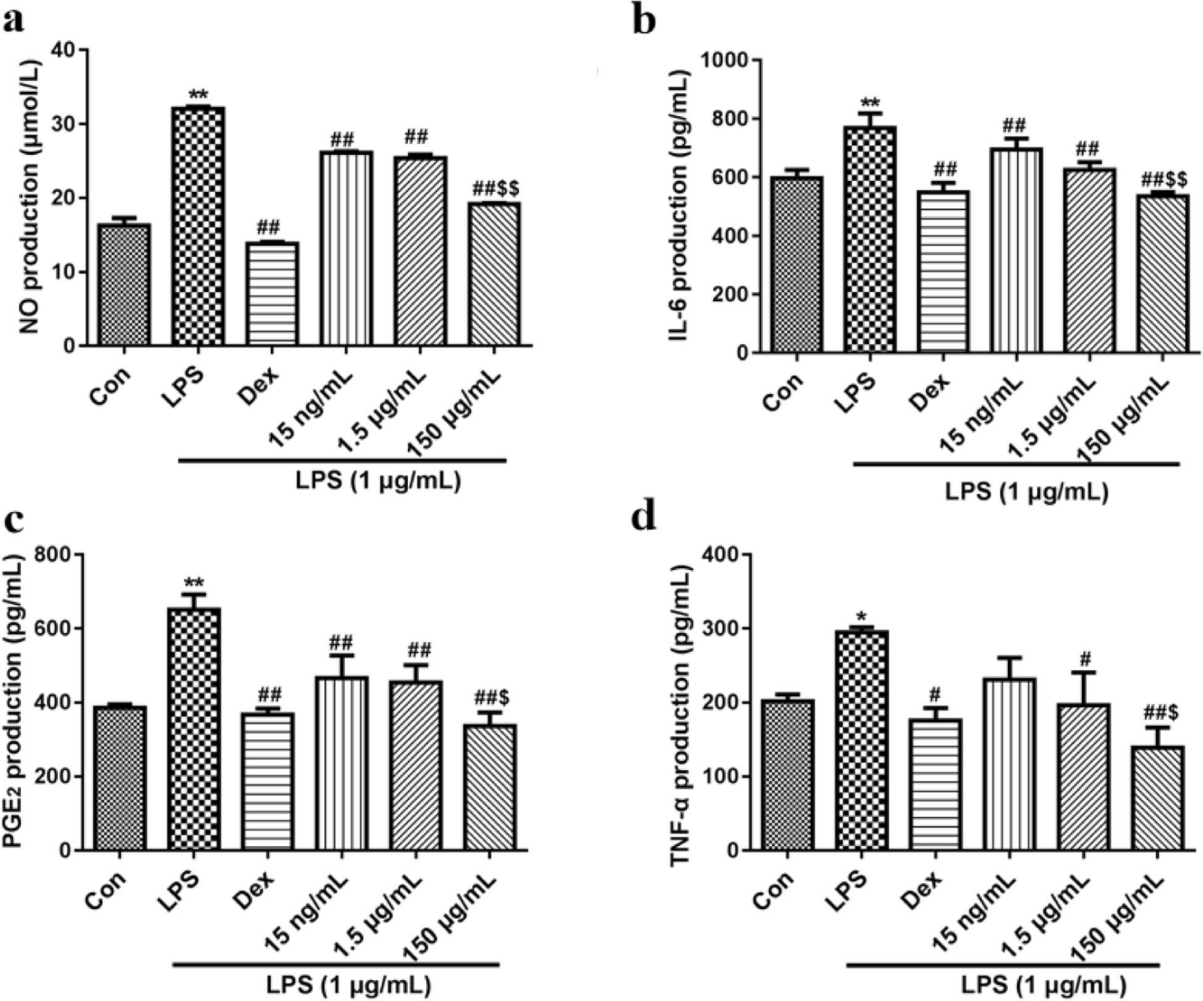
Effects on inflammatory factors in LPS-induced RAW264.7 cells. Cells were incubated with CSSPW (15 ng/mL, 1.5 μg/mL and 150 μg/mL), Dex (0.13 mg/mL) and LPS (1 μg/mL) for 24 h. The CSSPW could decrease the level of NO (**a**) to varying degrees. Similarly, LPS-induced increase in IL-6 (**b**), PGE_2_ (**c**) and TNF-α (**d**) contents were also inhibited after administration with CSSPW, especially at 150 μg/mL. All data were represented as mean ± SD (*n* = 6). ^*^p < 0.05, ^**^p < 0.01 vs. the Con group; ^*#*^*p* < 0.05, ^##^*p* < 0.01 vs. the LPS group; ^$^*p* < 0.05, ^$$^*p* < 0.01 vs. the 15 ng/mL group

It is accepted that the inflammatory process leads to the production of considerable inflammatory factors. Thus, to investigate the anti-inflammatory activity of CSSPW, we examined the effects of CSSPW on the levels of NO, IL-6, TNF-α and PGE_2_ in LPS-induced RAW264.7 cells. As presented in Fig. 3a-d, treatment with LPS significantly promoted the production of NO, TNF-α, IL-6 and PGE_2_ (*p* < 0.05 or *p* < 0.01), whereas CSSPW treatment particularly suppressed this increase in a concentration-dependent manner. In particular, the increased production was inhibited more effectively at 150 μg/mL than at 15 ng/mL (*p* < 0.05 or *p* < 0.01); however, there were no significant differences at 1.5 μg/mL.

### Effects on the expression levels of COX-2 and iNOS mRNA

**Fig. 4:**
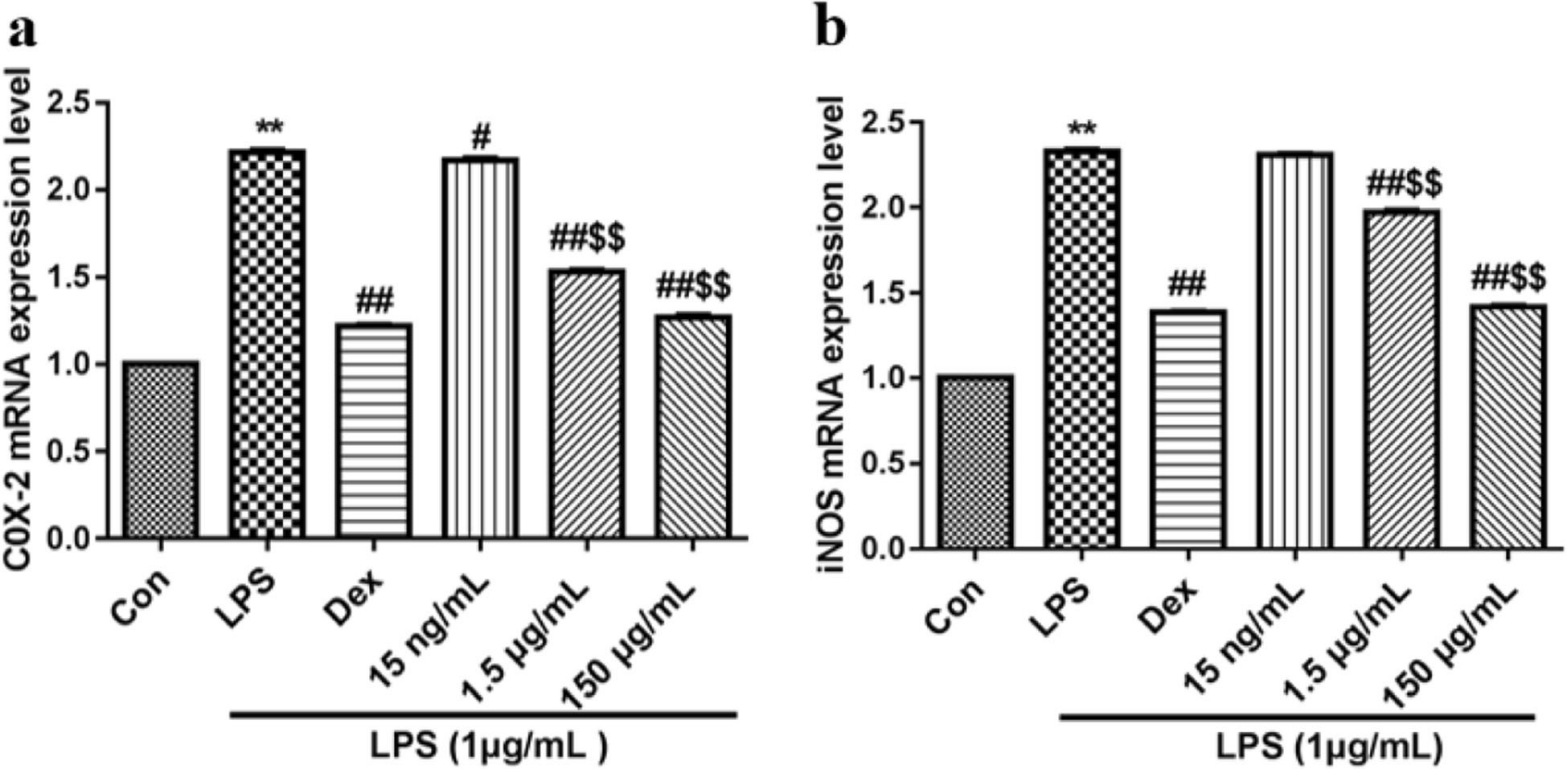
Effects of CSSPW on mRNA expression levels of COX-2 (**a**), iNOS (**b**) in LPS-induced RAW264.7 cells. Cells were incubated with CSSPW (15 ng/mL, 1.5 μg/mL and 150 μg/mL), Dex (0.13 mg/mL) and LPS (1 μg/mL) for 24 h. COX-2 and iNOS mRNA expression levels were detected by qPCR. Data were represented as mean ± SD (*n* = 6). ^**^p < 0.01 vs. the Con group; ^#^*p* < 0.05, ^##^*p* < 0.01 vs. the LPS group; ^$$^*p* < 0.01 vs. the 15 ng/mL group

Furthermore, we determined whether the inhibition of PGE_2_ and NO by CSSPW was due to downregulating the expression of COX-2 and iNOS mRNA. As expected, the expression levels of COX-2 and iNOS mRNA were obviously increased due to LPS stimulation (*p* < 0.01). Different concentrations of CSSPW caused a decrease in the expression of COX-2 and iNOS mRNA to varying degrees. The 1.5 μg/mL and 150 μg/mL groups had more significant effects on the expression levels of COX-2 and iNOS mRNA compared to those of the 15 ng/mL group (*p* < 0.01), especially those of the 150 μg/mL group (Fig. 4). These results indicated that CSSPW may suppress LPS-induced PGE_2_ and NO production by downregulating COX-2 and iNOS mRNA expression in RAW264.7 cells.

### Effects on protein expression

#### Nrf2/HO-1 pathway

**Fig. 5:**
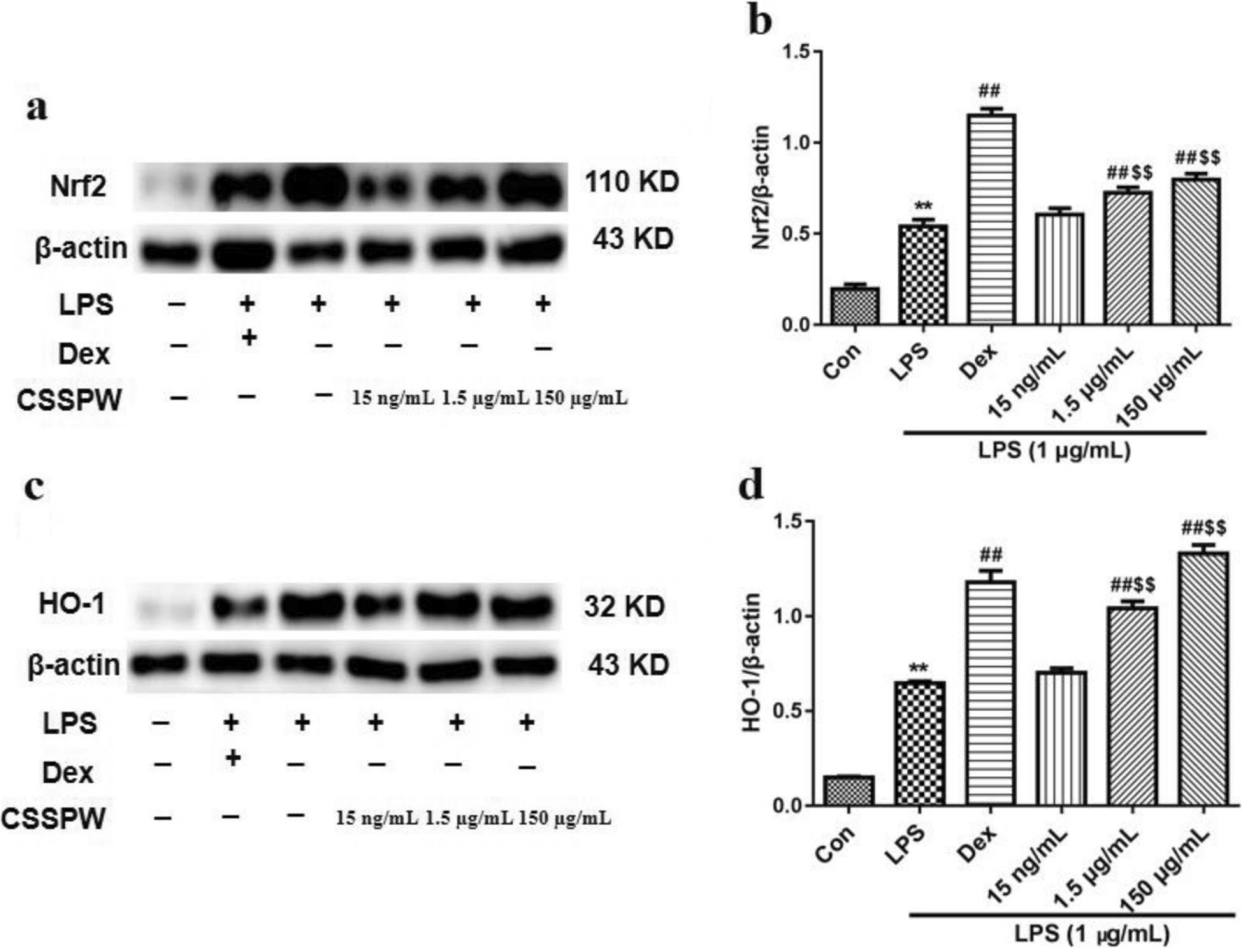
Effects of CSSPW on the expression of Nrf2/HO-1 pathway in LPS-induced RAW264.7 cells. Cells were incubated with CSSPW (15 ng/mL, 1.5 μg/mL and 150 μg/mL), Dex (0.13 mg/mL) and LPS (1 μg/mL) for 24 h. **a** and **c** Western blotting results of Nrf2 and HO-1 proteins. Graphs represented relative expression of Nrf2 (**b**) and HO-1 (**d**). Data were represented as mean ± SD (*n* = 3); ^**^p < 0.01 vs. the Con group; ^##^*p* < 0.01 vs. the LPS group; ^$$^*p* < 0.01 vs. the 15 ng/mL group

RAW264.7 cells exposed to LPS had increased expression of Nrf2 and HO-1 (*p* < 0.01). Interestingly, CSSPW at 1.5 μg/mL and 150 μg/mL further increased the expression levels of Nrf2 and HO-1 proteins (*p* < 0.01), whereas no obvious difference was found in the 15 ng/mL group (*p* > 0.05, Fig. 5).

#### MAPK pathway

**Fig. 6:**
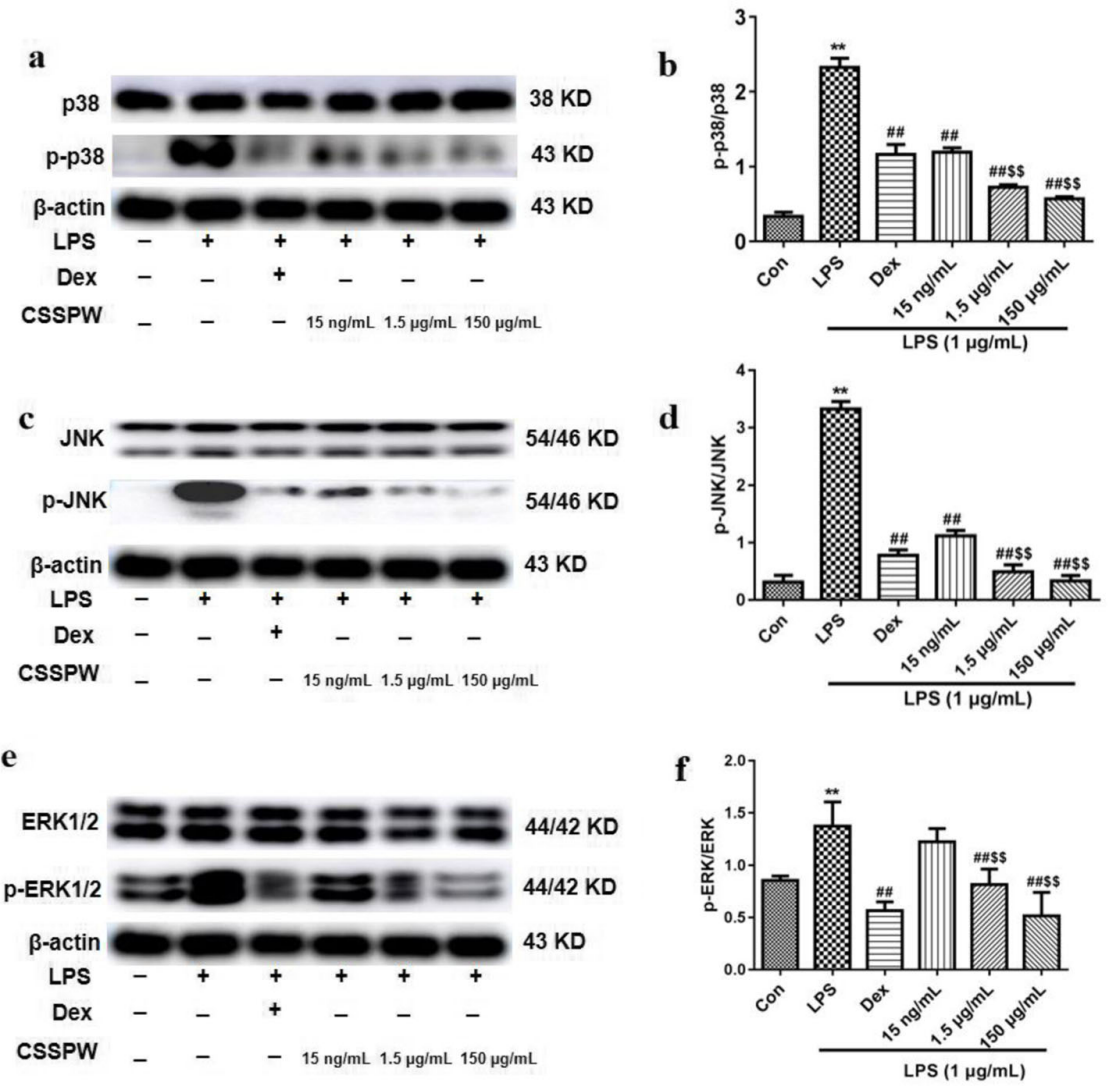
Effects of CSSPW on the expression of MAPK pathway in LPS-induced RAW264.7 cells. Cells were incubated with CSSPW (15 ng/mL, 1.5 μg/mL and 150 μg/mL), Dex (0.13 mg/mL) and LPS (1 μg/mL) for 24 h. **a**, **c**, **e** Western blotting results of MAPK pathway. Graphs represented relative expression of p-p38/p-38 (**b**), p-JNK/JNK (**d**), p-ERK/ERK (**f**). Data were represented as mean ± SD (*n* = 3); ^**^p < 0.01 vs. the Con group; ^##^*p* < 0.01 vs. the LPS group; ^$$^*p* < 0.01 vs. the 15 ng/mL group

Treatment with LPS resulted in a significant increase in the phosphorylation of p38, JNK and ERK compared to that of the Con group (*p* < 0.01). The different concentrations of CSSPW greatly reduced the expression levels of these proteins (*p* < 0.01). The effects in the 1.5 μg/mL and 150 μg/mL groups were more significant compared to those of the 15 ng/mL group (*p* < 0.01), especially those of the 150 μg/mL group (Fig. 6).

#### NF-κB pathway

**Fig. 7:**
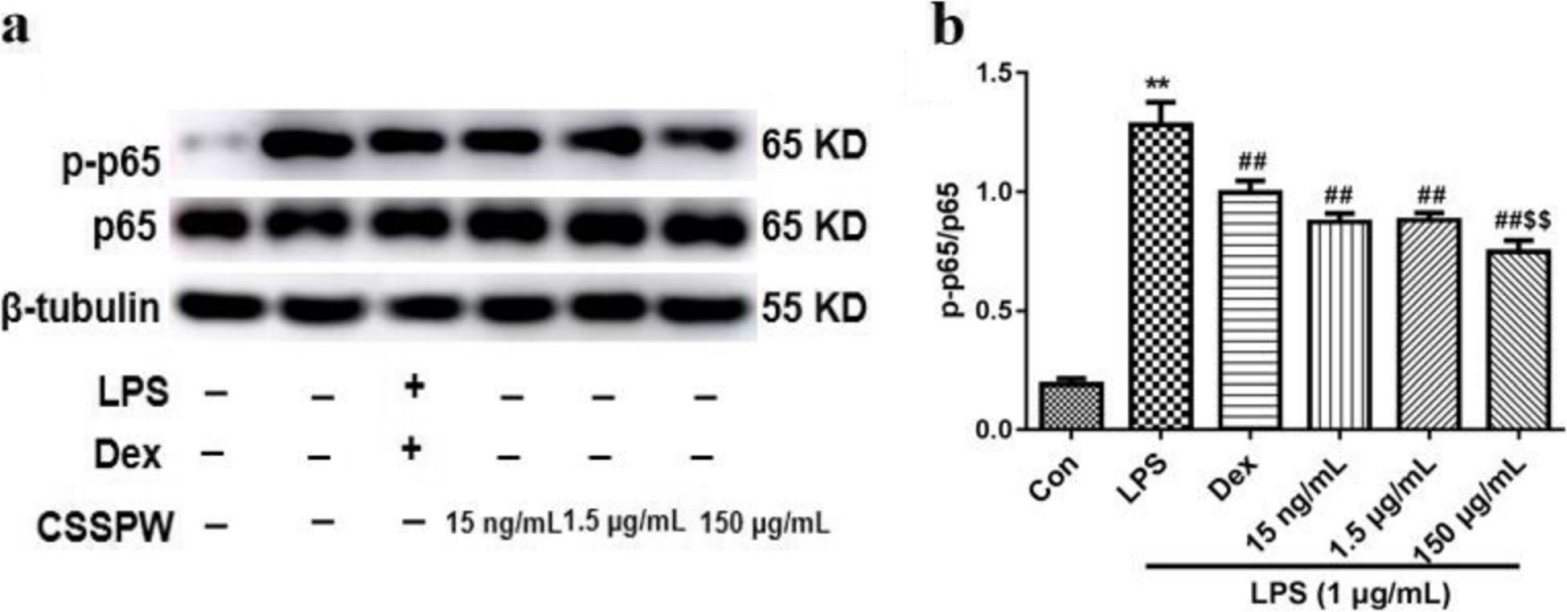
Effects of CSSPW on the expression of NF-κB pathway in LPS-induced RAW264.7 cells. Cells were incubated with CSSPW (15 ng/mL, 1.5 μg/mL and 150 μg/mL), Dex (0.13 mg/mL) and LPS (1 μg/mL) for 24 h. **a** Western blotting results of NF-κB pathway. **b** Graphs represented relative expression of p-p65/p65. All data were represented as mean ± SD (*n* = 3);^**^p < 0.01 vs. the Con group; ^##^*p* < 0.01 vs. the LPS group; ^$$^*p* < 0.01 vs. the 15 ng/mL group

Figure 7 showed that LPS stimulation resulted in an extreme increase in the phosphorylation of p65 compared to that of the Con group (*p* < 0.01), which was attenuated by CSSPW at different concentrations (*p* < 0.01). Moreover, CSSPW (150 μg/mL) treatment markedly suppressed the phosphorylation of p65 in LPS-stimulated RAW264.7 cells compared to that of the 15 ng/mL group (*p* < 0.01); however, there was no significant difference in the 1.5 μg/mL group (*p* > 0.05).

### Effects on p65 translocation into the nucleus

**Fig. 8:**
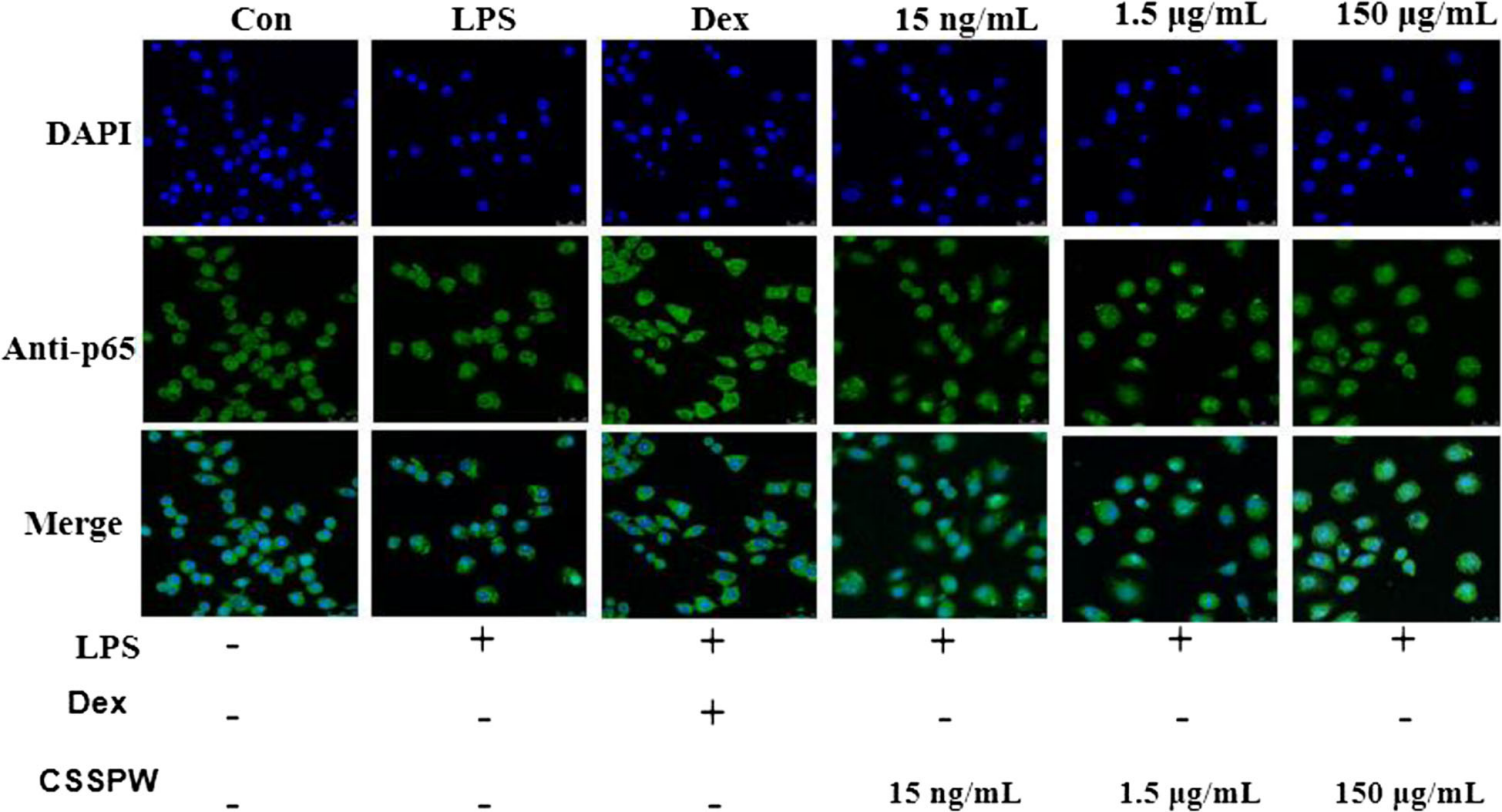
Effects of CSSPW on the activation of p65 into the nucleus in LPS-induced RAW264.7 cells (× 630). Cells were incubated with CSSPW (15 ng/mL, 1.5 μg/mL and 150 μg/mL), Dex (0.13 mg/mL) and LPS (1 μg/mL) for 24 h. The nuclear translocation of p65 was detected by Immunofluorescence. Nuclei were stained by DAPI (blue). The low proportion of p65 into the nucleus represented a better anti-inflammatory effect

Under normal conditions, p65 was mainly distributed in the cytoplasm. After LPS stimulation, significant green fluorescence was observed in the nuclear region. As shown in Fig. 8, correspondingly, the proportion of p65 in the nucleus following LPS treatment was greatly increased compared to that of the Con group. Interestingly, the addition of CSSPW inhibited the nuclear translocation of p65 with increasing concentrations of the drug, the proportion of p65 into nucleus was decreased.

### Effects on ROS production

**Fig. 9:**
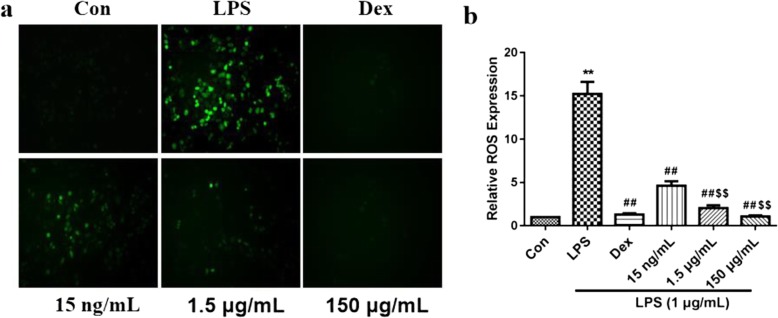
Effects of CSSPW on the expression of ROS in LPS-induced RAW264.7 cells (200 ×). Cells were incubated with CSSPW (15 ng/mL, 1.5 μg/mL and 150 μg/mL), Dex (0.13 mg/mL) and LPS (1 μg/mL) for 24 h. **a** ROS expression result. **b** Graphs represented ROS expression rate. Fluorescent probe was used to detect the expression level of ROS. Data were represented as mean ± SD (*n* = 3). ^**^p < 0.01 vs. the Con group; ^##^*p* < 0.01 vs. the LPS group; ^$$^*p* < 0.01 vs. the 15 ng/mL group

ROS content was detected by the fluorescent probe DCFH-DA. As demonstrated in Fig. 9, ROS was increased in LPS-treated macrophages (*p* < 0.01), whereas LPS-induced ROS generation was significantly inhibited by CSSPW treatment (*p* < 0.01). Moreover, the effects in the 150 μg/mL and 1.5 μg/mL groups were better than that in the 15 ng/mL group (*p* < 0.01), especially that in the 150 μg/mL group.

### UV fingerprint analysis

**Fig. 10:**
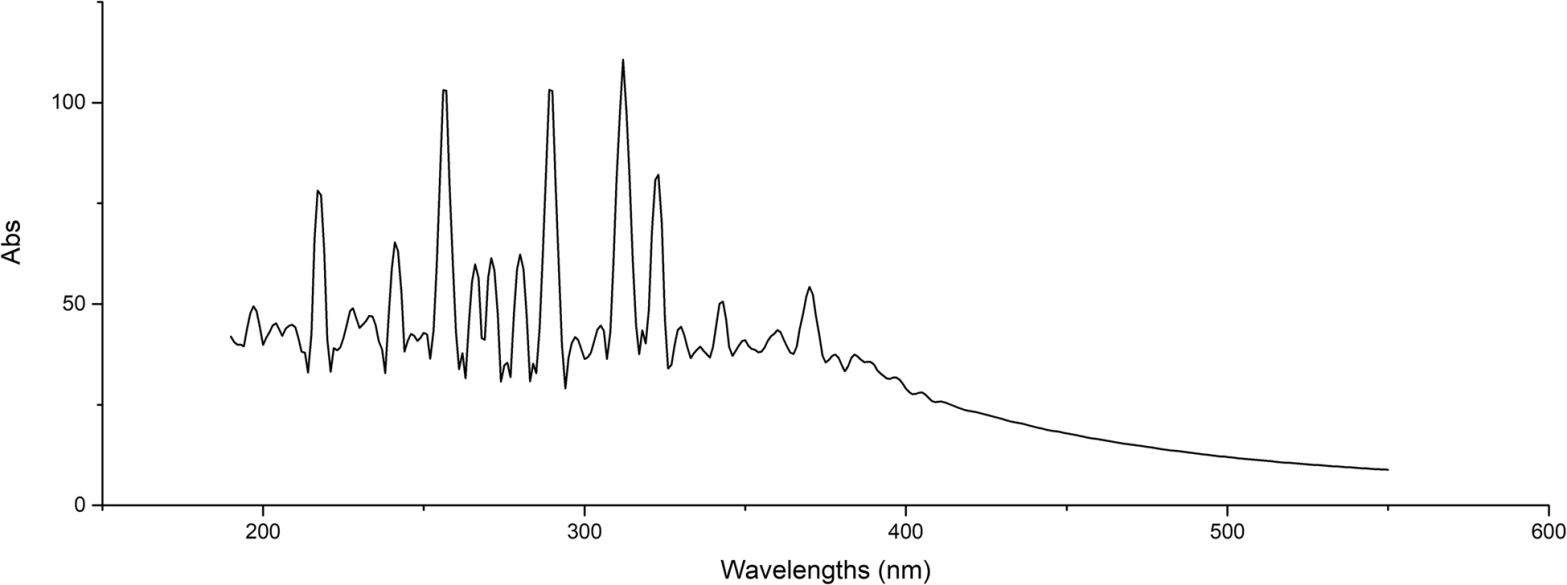
UV fingerprint of CSSPW from *C. serratus*. It had absorption at characteristic wavelengths

As could be seen from the full-wavelength scanning spectrum of the sample (Fig. 10), the main absorption range was 200–400 nm. CSSPW had absorption peaks at 367 nm, 359 nm, 323 nm, 301 nm, 280 nm and 259 nm. That was, it had absorption at characteristic wavelengths, and there was no interference in the range of visible light.

## Discussion

Inflammation can occur in tissues and organs in different parts of the body, due to conditions such as pneumonia, hepatitis and nephritis. Excessive inflammatory factors produced during inflammation can damage the body directly or indirectly [13]. In this study, RAW264.7 macrophages were used as the research object to elucidate the effect of CSSPW on inflammatory factors and mediators as well as related proteins, thus confirming the anti-inflammatory effect of CSSPW.

Activated RAW264.7 macrophages are involved in many inflammatory responses in the body. Inflammation serves a key role in the host defence system. The bacterial endotoxin LPS stimulates a powerful inflammatory signal that contributes to increased monocyte/macrophage activation and expression of pro-inflammatory cytokines (NO, TNF-α, IL-6, PGE_2_, iNOS and COX-2) and pathways (NF-κB, MAPK and Nrf2/HO-1) [14], thereby mediating inflammatory responses [15].

COX-2 and iNOS are key enzymes that promote the generation of PGE_2_ and NO, respectively, and both can be induced by LPS or other cytokines [16]. The expression levels of COX-2 and iNOS mRNA reflect the degree of cell damage and play synergistic roles in a variety of inflammatory diseases [17]. PGE_2_ is a classic inflammatory mediator, and its increase leads to the continuation of inflammatory reactions [18]. Inhibiting COX-2 mRNA expression inhibits the production of PGE_2_, thereby reducing inflammatory damage [19, 20]. NO changes the permeability of the cell membrane to promote the release of inflammatory factors such as TNF-α, IL-6 and PGE_2_, which plays a key role in the occurrence and development of inflammation [21, 22]. TNF-α, IL-6 and PGE_2_ are common inflammatory factors that reflect the severity of inflammation [23]. It has been reported that LPS promotes the synthesis and release of TNF-α, IL-6 and PGE_2_, thereby aggravating the inflammatory response [24]. Therefore, reducing the levels of inflammatory factors (such as NO, TNF-α, IL-6 and PGE_2_), as well as inhibiting the expression of COX-2 and iNOS mRNA, are important measures to prevent inflammatory response [6]. The present study demonstrated that CSSPW could significantly reduce the production of NO, TNF-α, IL-6 and PGE_2,_ as well as the expression levels of COX-2 and iNOS mRNA in a dose-dependent manner, suggesting that CSSPW may exert an anti-inflammatory effect by inhibiting pro-inflammatory cytokines and mediators. However, we cannot exclude the involvement of other pro-inflammatory cytokines as a possible factor.

Inflammation promotes ROS production, and the over-expression of ROS leads to lipid peroxidation, which may cause serious cell damage. An increase in ROS content is often considered an important indicator of body damage [25]. This study found that CSSPW could inhibit the LPS-induced formation of ROS in a dose-dependent manner, indicating that CSSPW has an anti-inflammatory activity. ROS can also trigger the NF-κB and MAPK pathways, as well as promote the expression of inflammatory factors such as NO, TNF-α, IL-6 and PGE_2_, thus exacerbating the inflammatory response [26].

The NF-κB pathway is generally considered to be a key inflammatory signalling pathway, which is involved in immune and inflammatory responses in vivo, including apoptosis, cell adhesion, cytokine production, etc. [17]. Growing evidence suggests that the NF-κB pathway mediates inflammatory processes by regulating the expression of the inflammatory response genes (COX-2 and iNOS mRNA) and various pro-inflammatory cytokines [27]. p65, a member of the NF-κB protein family, is activated to p-p65 under LPS stimulation, resulting in nuclear translocation and the production of various pro-inflammatory factors (TNF-α, IL-6, COX-2, iNOS, etc.), thereby accelerating the inflammatory process [28, 29]. Hence, the expression of p65 and p-p65 can effectively reflect the degree of NF-κB activation, and inhibiting the activation of the NF-κB pathway can exert anti-inflammatory effects [30, 31]. In this study, CSSPW inhibited LPS-induced p65 protein expression and p65 nuclear translocation in a dose-dependent manner, suggesting that anti-inflammatory effects may be exerted by mediating the NF-κB pathway.

The MAPK pathway can regulate the synthesis or release of pro-inflammatory factors (NO, TNF-α and IL-6) and plays an important role in the inflammatory response [32]. The JNK, p38 and ERK pathways are the three main pathways of the MAPK pathway family and are involved in the inflammatory response; these proteins can be activated and phosphorylated by LPS, thus regulating the downstream inflammatory cytokines [33]. Thus, inhibiting the activation of the MAPK pathway can inhibit the production of NO, TNF-α and IL-6, as well as the expression of COX-2 and iNOS mRNA, further exerting anti-inflammatory effects [34]. In this study, a certain concentration of CSSPW significantly inhibited LPS-induced activation of the p38, JNK and ERK pathways, indicating that CSSPW has anti-inflammatory activity. Furthermore, in a certain concentration range, higher concentrations of CSSPW exerted more obvious anti-inflammatory effects. These results suggest that the MAPK pathway may be involved in the inhibitory effect of CSSPW on LPS-stimulated RAW264.7 cells.

The Nrf2/HO-1 signalling pathway also plays a vital role in the process of inflammatory response. LPS can activate Nrf2 through a series of signalling molecules, as well as oxidative stress, and regulate the expression of downstream target proteins (such as HO-1) [32, 35]. The upregulation of Nrf2 can downregulate the production of ROS, thus inhibiting inflammatory responses [36]. HO-1 is a key molecule that regulates the inflammatory response and inhibits the production of pro-inflammatory mediators such as TNF-α and IL-6 [33, 37]. A typical response of cells and tissues to oxidative stress is upregulating the expression levels of Nrf2 and HO-1. For example, ginkgolide exerts its anti-inflammatory activity by upregulating the expression of Nrf2 and HO-1 [38]. In this study, CSSPW at doses of 1.5 and 150 μg/mL could further significantly increase the expression of Nrf2 and HO-1 to exert an anti-inflammatory effect; in contrast, the effect of 15 ng/mL CSSPW was insignificant, suggesting a dose-dependent effect.

## Conclusions

In summary, the current study demonstrated that CSSPW may exert anti-inflammatory activity in a dose-dependent manner by suppressing the NF-κB and MAPK inflammatory pathways and activating the Nrf2/HO-1 pathway to reduce the release of inflammatory factors such as NO, TNF-α, IL-6 and PGE_2_ in LPS-stimulated macrophages. To the best of our knowledge, this is the first report of the anti-inflammatory activity of *C. serratus* in LPS-induced cells*.* These results provide evidence for the clinical treatment of inflammatory diseases, indicating that it is possible to develop CSSPW into an effective anti-inflammatory medicine. Furthermore, we will test the phosphorylation level of IκB protein in the NF-κB pathway and the phosphorylation of Nrf2 protein in the Nrf2/HO-1 pathway. In the future, we expect to isolate the anti-inflammatory active monomer composition of *C. serratus.*

## Data Availability

The data presented in this manuscript has not been deposited in any repository yet. However, the materials are available to the researchers upon request.

## Abbreviations

*C. serratus*: *Chloranthus serratus*
COX-2: Cyclooxygenase-2
CSSPW: *Chloranthus serratus*’s separated part of water
DMEM: Dulbecco’s modified Eagle’s medium
IL-6: Interleukin-6
iNOS: Inducible nitric oxide synthase
LPS: Lipopolysaccharide
MAPK: Mitogen-activated protein kinase
NF-κB: Nuclear factor-kappa B
NO: Nitric oxide
Nrf2/HO-1: Nuclear transcription factor E2 related factor 2/heme oxygenase-1
PGE_2_: ProstaglandinE_2_
qPCR: Real-time quantitative PCR
ROS: Reactive oxygen species
TCM: Traditional Chinese medicine
TNF-α: Tumor necrosis factor-α

## References

[1] Huang GJ, Huang SS, Deng JS (2012). Anti-inflammatory activities of inotilone from *Phellinus linteus* through the inhibition of MMP-9, NF-κB, and MAPK activation *in vitro* and *in vivo*. PLoS One.

[2] Jang JY, Lee MJ, You BR, Jin JS, Lee SH, Yun YR, Kim HJ (2017). *Allium hookeri* root extract exerts anti-inflammatory effects by nuclear factor-κB down-regulation in lipopolysaccharide-induced RAW264.7 cells. BMC Complement Altern Med.

[3] Shin JY, Kang JS, Byun HW, Ahn EK (2017). Regulatory effects and molecular mechanism of *Trigonostemonreidioides* on lipopolysaccharide-induced inflammatory responses in RAW264.7 cells. Mol Med Rep.

[4] Mirzoeva S, Tong X, Bridgeman BB, Plebanek MP, Volpert OV (2018). Apigenin inhibits UVB-induced skin carcinogenesis: the role of thrombospondin-1 as an anti-inflammatory factor. Neoplasia.

[5] Chang SL, Hsiao YW, Tsai YN, Lin SF, Liu SH, Lin YJ, Lo LW, Chung FP, Chao TF, Hu YF, Tuan TC, Liao JN, Hsieh YC, Wu TJ, Higa S, Chen SA (2018). Interleukin-17 enhances cardiac ventricular remodeling via activating MAPK pathway in ischemic heart failure. J Mol Cell Cardiol.

[6] Hsieh IN, Chang ASY, Teng CM, Chen CC, Yang CR (2011). Aciculatin inhibits lipopolysaccharide-mediated inducible nitric oxide synthase and cyclooxygenase-2 expression via suppressing NF-κB and JNK/p38 MAPK activation pathways. J Biomed Sci.

[7] Liang N, Sang Y, Liu W, Yu W, Wang X (2018). Anti-inflammatory effects of Gingerol on lipopolysaccharide-stimulated RAW264.7 cells by inhibiting NF-κB signaling pathway. Inflammation.

[8] Marilene LDR, Leandra EGO, Camila CMPS, Damiao PDS, Reinaldo NDA, Demetrius AMA (2013). Antinociceptive and anti-inflammatory effects of the monoterpene α, β-epoxy-carvone in mice. J Nat Med.

[9] Zhang M, Iinuma M, Wang JS, Oyama M, Ito T, Kong LY (2012). Terpenoids from *Chloranthus serratus* and their anti-inflammatory activities. J Nat Prod.

[10] Song L. Modern Chinese pharmacy dictionary, vol. 1: People’s Health Publishing House; 2001. p. 222–3. https://www.ncbi.nlm.nih.gov/pubmed/22372956.

[11] Sun SP, Li SL (2013). Study on anti-inflammatory effect of alcohol extract of *Chloranthus serratus*. Chinese Mod Med Distance Educ.

[12] Su XD, Guo RH, Li HX, Ma JY, Kim YR, Kim YH, Yang SY (2018). Anti-allergic inflammatory components from *Sanguisorba officinalis* L. Bioorg Med Chem Lett.

[13] George A, Chinnappan S, Chintamaneni M, Kotak CV, Choudhary Y, Kueper T, Radhakrishnan AK (2014). Anti-inflammatory effects of Polygonum minus (Huds) extract (Lineminus™) in *in-vitro* enzyme assays and carrageenan induced paw edema. BMC Complement Altern Med.

[14] Sun HJ, Cai WW, Wang X, Liu YL, Hou B, Zhu XX, Qiu LY (2017). Vaccaria hypaphorine alleviates lipopolysaccharide-induced inflammation via inactivation of NFκB and ERK pathways in raw 264.7 cells. BMC Complement Altern Med.

[15] Choi HE, Kwak HJ, Kim SK, Cheon HG (2018). Foenumoside B isolated from Lysimachiafoenum-graecum extract suppresses LPE-induced inflammatory response via NF-κB/AP-1 inactivation in murine macrophages and in endotoxin-induced shock model. Eur J Pharmacol.

[16] Lee KH, Chow YL, Sharmili V, Abas F, Alitheen NBM, Shaari K, Israf DA, Lajis NH, Syahida A (2012). A Curcumin derivative suppresses inflammatory responses in macrophage-like cellular system: role of inhibition in NF-κB and MAPK signaling pathways. Int J Mol Sci.

[17] Yoon WJ, Moon JY, Song G, Lee YK, Han MS, Lee JS, Ihm BS, Lee WJ, Lee NH, Hyun CG (2010). Artemisia fukudo essential oil attenuates LPS-induced inflammation by suppressing NF-κB and MAPK activation in RAW264.7 macrophages. Food Chem Toxicol.

[18] Li T, Liu B, Mao W, Gao R, Wu J, Deng Y, Shen Y, Liu K, Cao J (2019). Prostaglandin E promotes nitric oxide synthase 2, platelet-activating factor receptor, and matrix metalloproteinase-2 expression in Escherichia coli-challenged *ex vivo* endometrial explants via the prostaglandin E receptor 4/protein kinase a signaling pathway. Theriogenology.

[19] Albertini R, Aimbire F, Villaverde AB, Sliva JA, Costa MS (2007). COX-2 mRNA expression decreases in the subplantar muscle of rat paw subjected to carrageenan-induced inflammation after low level laser therapy. Inflamm Res.

[20] Song Q, Fan C, Wang P, Li Y, Yang M, Yu SY (2018). Hippocampal CA1 βCaMKII mediates neuroinflammatory responses via COX-2/PGE_2_ signaling pathways in depression. J Neuroinflammation.

[21] Lee YJ, Choi DY, Choi IS, Kim KH, Kim YH, Kim HM, Lee K, Cho WG, Jung JK, Han SB, Han JY, Nam SY, Yun YW, Jeong JH, Oh KW, Hong JT (2012). Inhibitory effect of 4-O-methylhonokiol on lipopolysaccharide-induced neuroinflammation, amyloidogenesis and memory impairment via inhibition of nuclear factor-kappaB *in vitro* and *in vivo* models. J Neuroinflammation.

[22] Lee PJ, Tsai TY, Chen S (2018). Analysis of NO-suppressing activity of strawberry wine supplemented with ball-milled achenes. J Food Sci Technol.

[23] Badiei A, Muniraj N, Chambers S, Bhatia M (2014). Inhibition of hydrogen sulfide production by gene silencing attenuates inflammatory activity by down-regulation of NF-κB and MAPK-inase activity in LPS-activated RAW 264.7 cells. Biomed Res Int.

[24] Lee WH, Wu HM, Lee CG, Sung DI, Song HJ, Matsui T, Kim HB, Kim SG (2014). Specific Oligopeptides in *Fermented Soybean* extract inhibit NF-κB-dependent iNOS and cytokine induction by toll-like receptor ligands. J Med Food.

[25] Ihsan AU, Khan FU, Khongorzul P, Ahmad KA, Naveed M, Yasmeen S, Cao Y, Taleb A, Maiti R, Akhter F, Liao X, Li X, Cheng Y, Khan HU, Alam K, Zhou X (2018). Role of oxidative stress in pathology of chronic prostatitis/chronic pelvic pain syndrome and male infertility and antioxidants function in ameliorating oxidative stress. Biomed Pharmacother.

[26] Younis T, Khan MR, Sajid M, Majid M, Zahra Z, Shah NA (2016). Fraxinus xanthoxyloides leaves reduced the level of inflammatory mediators during *in vitro* and *in vivo* studies. BMC Complement Altern Med.

[27] Yodkeeree S, Ooppachai C, Pompimon W, Limtrakul P (2018). O-Methylbulbocapnine and Dicentrinesuppress LPS-induced inflammatory response by blocking NF-κB and AP-1 activation through inhibiting MAPKs and Aktsignaling in RAW264.7 macrophages. Biol Pharm Bull.

[28] Li Y, Liu H, Xu QS, Du YG, Xu J (2014). Chitosan oligosaccharides block LPS-induced O-GlcNAcylation of NF-κB and endothelial inflammatory response. Carbohydr Polym.

[29] Kang NJ, Han SC, Kang GJ, Koo DH, Koh YS, Hyun JW, Lee NH, Ko MH, Kang HK, Yoo ES (2015). Diphlorethohydroxycarmalol inhibits interleukin-6 production by regulating NF-κB, STAT5 and SOCS1 in lipopolysaccharide-stimulated RAW264.7 cells. Mar Drugs.

[30] Lee SY, Lee KS, Yi SH, Kook SH, Lee JC (2013). Acteoside suppresses RANKL-mediated osteoclastogenesis by inhibiting c-Fosinduction and NF-κB pathway and attenuating ROS production. PLoS One.

[31] Yang L, Chen J, Han X, Zhang E, Huang X, Guo X, Chen Q, Wu W, Zheng G, He D, Zhao Y, Yang Y, He J, Cai Z (2018). Pirh2 mediates the sensitivity of myeloma cells to bortezomib via canonical NF-κB signaling pathway. Protein Cell.

[32] Ye J, Piao HM, Jiang JZ, Jin GY, Zheng MY, Yang JS, Jin X, Sun T, Choi YH, Li LC, Yan GH (2017). Polydatin inhibits mast cell-mediated allergic inflammation by targeting PI3K/Akt, MAPK, NF-κB and Nrf2/HO-1 pathways. Sci Rep.

[33] Kim KS, Cui X, Lee DS, Ko W, Sohn JH, Yim JH, An RB, Kim YC, Oh H (2014). Inhibitory effects of benzaldehyde derivatives from the marine *Fungus Eurotium* sp. SF-5989 on inflammatory mediators via the induction of Heme Oxygenase-1 in lipopolysaccharide-stimulated RAW264.7 macrophages. Int J Mol Sci.

[34] Song S, Dang M, Kumar M. Anti-inflammatory and renal protective effect of gingerol in high-fat diet/streptozotocin-induced diabetic rats via inflammatory mechanism. Inflammopharmacology. 2019:1–12. https://sci-hub.tw/10.3390/ijms151223749. 10.1007/s10787-019-00569-6 30826930

[35] Zhang W, Song JK, Yan R, Li L, Xiao ZY, Zhou WX, Wang ZZ, Xiao W, Du GH (2018). Diterpene ginkgolides protect against cerebral ischemia/reperfusion damage in rats by activating Nrf2 and CREB through PI3K/Akt signaling. Acta Pharmacol Sin.

[36] Lv Q, Wang K, Qiao SM, Dai Y, Wei ZF (2018). Norisoboldine, a natural aryl hydrocarbon receptor agonist, alleviates TNBS-induced colitis in mice, by inhibiting the activation of NLRP3 inflammasome. Chin J Nat Med.

[37] Jin CH, So YK, Han SN, Kim JB (2016). Isoegomaketoneupregulates Heme Oxygenase-1 in RAW264.7 cells via ROS/p38 MAPK/Nrf2 pathways. Biomol Ther.

[38] Huang S, Meng N, Chang BQ, Quan XH, Yuan RY, Li B (2018). Anti-inflammatory activity of *Epimedium brevicornu Maxim* ethanol extract. J Med Food.

